# Combined experimental and computational study of high-pressure behavior of triphenylene

**DOI:** 10.1038/srep25600

**Published:** 2016-05-10

**Authors:** Xiao-Miao Zhao, Guo-Hua Zhong, Jiang Zhang, Qiao-Wei Huang, Alexander F. Goncharov, Hai-Qing Lin, Xiao-Jia Chen

**Affiliations:** 1 Department of Physics, South China University of Technology, Guangzhou 510640, China; 2 Center for High Pressure Science and Technology Advanced Research, Shanghai 201203, China; 3 Geophysical Laboratory, Carnegie Institution of Washington, Washington, D.C. 20015, USA; 4 Shenzhen Institutes of Advanced Technology, Chinese Academy of Sciences, Shenzhen 518055, China; 5 Beijing Computational Science Research Center, Beijing 100084, China

## Abstract

We have performed measurements of Raman scattering, synchrotron x-ray diffraction, and visible transmission spectroscopy combined with density functional theory calculations to study the pressure effect on solid triphenylene. The spectroscopic results demonstrate substantial change of the molecular configuration at 1.4 GPa from the abrupt change of splitting, disappearance, and appearance of some modes. The structure of triphenylene is found be to stable at high pressures without any evidence of structural transition from the x-ray diffraction patterns. The obtained lattice parameters show a good agreement between experiments and calculations. The obtained band gap systematically decreases with increasing pressure. With the application of pressure, the molecular planes become more and more parallel relative to each other. The theoretical calculations indicate that this organic compound becomes metallic at 180 GPa, fueling the hope for the possible realization of superconductivity at high pressure.

Polycyclic aromatic hydrocarbons (PAHs), that show extensive *π*-conjugation, have attracted much attention from scientific community because of the discovery of novel and interesting physical properties, such as metallization or superconductivity by doping alkali-metal. Motivated by the discovery of superconductivity in alkali doped picene with a superconducting transition temperature (*T*_*c*_) up to 18 K^1^, a series of superconductors based on phenanthrene (C_14_H_10_), coronene (C_24_H_12_), and 1,2:8,9-dibenzopentacene (C_30_H_18_) have been synthesized^2^,^3^,^4^,^5^. With increasing the length of the PAHs chains, the *T*_*c*_ was found to increase dramatically from 5 K to 33 K. In a theoretical study, Kato *et al.*^6^ reported that molecular edge structures as well as molecular sizes have relevance to the strength of electron-phonon coupling and *T*_*c*_s. Meanwhile, experimental studies have found that the edge structure, armchair or zigzag, of the organic molecule may play an important role in superconductivity of PAHs where the armchair edge is thought to be a key factor for superconductivity^4^. In pioneering study, Ginzburg^7^ suggested the possible realization of high-*T*_*c*_ and even room temperature superconductivity in organic materials through the nonphonon mechanism. In fact, the first organic superconductor was discovered in charge-transfer salts under pressure^8^. The application of pressure has also driven Cs_3_C_60_ from insulator to superconductor, with the highest *T*_*c*_ of 38 K in fullerides system^9^. Pressure also can enhance superconductivity in PAHs-based superconductors^2^. The *T*_*c*_ is doubled with application of pressure in K_3_picene (18 K phase) with an intial pressure derivation of *T*_*c*_, *dT*_*c*_/*dP* = 12.5 K/GPa^10^. It is clear that applying pressure is of paramount importance to explore superconductivity within organic hydrocarbon systems.

Extensive investigations on PAHs have showed that pentacene, a straight-chain aromatic hydrocarbon, exhibits metallic character with a positive temperature coefficient of resistance at 27 GPa^11^. In addition, benzene was predicted^12^ theoretically to enter a metallic phase at ~190 GPa. Although the experimental examination of the theoretical prediction is on the way, there are only a few high-pressure studies on these PAHs reported in the literature. It remains unclear whether superconductivity can be induced by only applying pressure in these materials, after entering their metallic state. We have chosen one of PAHs, triphenylene (C_18_H_12_) with armchair edge, to explore the details of its high-pressure behavior. Pure triphenylene possesses othorhombic symmetry with four molecules per unit cell, the space group *P*2_1_2_1_2_1_ and lattice parameters: *a* = 5.280(2) Å, *b* = 12.972(5) Å, *c* = 16.707(7) Å^13^. While the crystal structure and vibrational properties of this hydrocarbon material at ambient conditions are available^13^,^14^, such information is absent at high pressures. Previous theoretical studies^15^,^16^,^17^ suggest that the molecules with armchair edge would have larger electron-phonon coupling constants than those with acene-edge type hydrocarbons, which is large enough to account for the experimentally measured *T*_*c*_s. Triphenylene is being examined as a potential candidate along this direction for exploring its metallization and superconductivity. A thorough investigation focused on its high-pressure vibrational properties and crystal and electron structures is highly desired.

In this work, we performed high-pressure Raman scattering, synchrotron x-ray diffraction (XRD), and visible transmission spectroscopy measurements on triphenylene under pressure. We also optimized the internal geometry and calculated the density of states (DOS) based on the density-function theory (DFT) calculations. The combination of experiments and calculations allow us to examine the phase stability of this material upon compression. Our results show that triphenylene maintains its initial structure up to 200 GPa. At 180 GPa, the band gap closes and the material enters a metallic state.

## Results

### Evolution of vibrational properties with pressure

Raman scattering, which measures phonons (lattice and molecular vibrations) in Brillouin zone center, is a powerful technique to detect small structural distortion or the change of molecular configuration via the observation of the band splitting and/or soft modes. Experimental data about Raman spectra of triphenylene at ambient pressure are already available in the literature^14^. In the present study, we have collected the Raman spectra within the region from 100 to 2000 cm^−1^ as a function of pressure up to 30.4 GPa. A detailed list of peak frequency and modes assignment measured at ambient condition is shown in Table 1. We found that our measured spectra at ambient pressure are in a good agreement with those in the literature. On the basis of analysis of the Raman spectra, we found that the vibrational properties of the studied material are very similar to other parent PAHs^18^,^19^,^20^,^21^.

**Figure 1:**
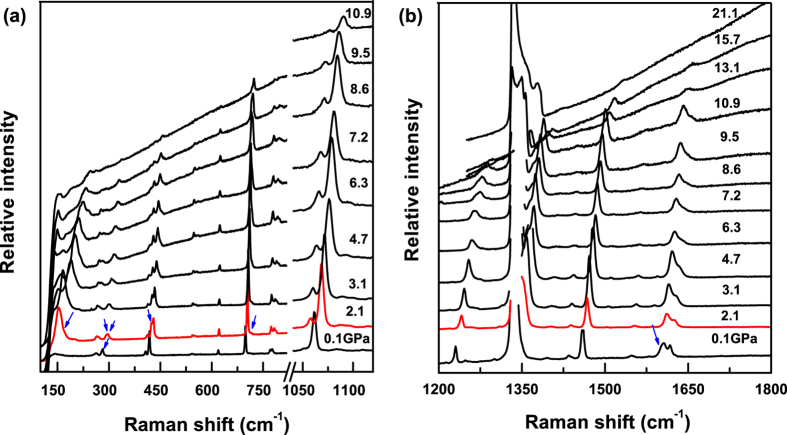
Representative Raman spectra of triphenylene in low frequency regions (**a**) and high frequency regions (**b**) under different hydrostatic pressure in the backscattering geometry. The blue arrows mark the changes of part Raman modes to make them clearer.

The Raman spectra of triphenylene at various pressures up to 21.1 GPa at room temperature are shown in Fig. 1. Clearly, the Raman spectra could be divided into two regions shown in Fig. 1(a,b). When the pressure is increased up to 2.1 GPa, we can clearly see that the spectra are different with those at ambient pressure. Several qualitative features of the Raman spectra in the triphenylene can be discerned for increasing pressure. In the low frequency, it is worth noting that the three modes of *ν*_1_, *ν*_2_ and *ν*_4_, corresponding to C-C-C out of plane bending, split into two modes. Furthermore, a shoulder peak appears on the high-frequency side of *ν*_7_, and the intensity of the vibrational mode gradually increases with increasing pressure. It is noteworthy to mention that the adjacent two vibration modes of *ν*_25_ and *ν*_26_, associated with C-C stretching, combine into a peak. In addition, the shoulder peak located in the low-frequency side of *ν*_30_ disappears as pressure is increased up to 2.1 GPa. These features in the Raman spectra suggest substantial changes in the molecular configuration or crystal structures above 2.1 GPa. When looking at the evolution of the Raman spectra, most vibrational modes shift toward higher frequencies by applied pressure. With a further increase of pressure up to 10.9 GPa, most of low frequency modes shown in Fig. 1(a) disappear, which may be due to the generation of fluorescence, and may also be due to the changes of crystal structures. For the higher frequency region, the vibrational modes display a smooth and uniform hardening with pressure up to 30.4 GPa without any obvious changes. We only show the Raman spectra of pressure up to 21.1 GPa in Fig. 1 due to the weak signal at higher pressures. The whole Raman spectra of triphenylene do not show abrupt changes except some subtle changes, which is very similar to the high-pressure behavior of picene, indicating the stability of triphenylene at high pressures.

**Figure 2:**
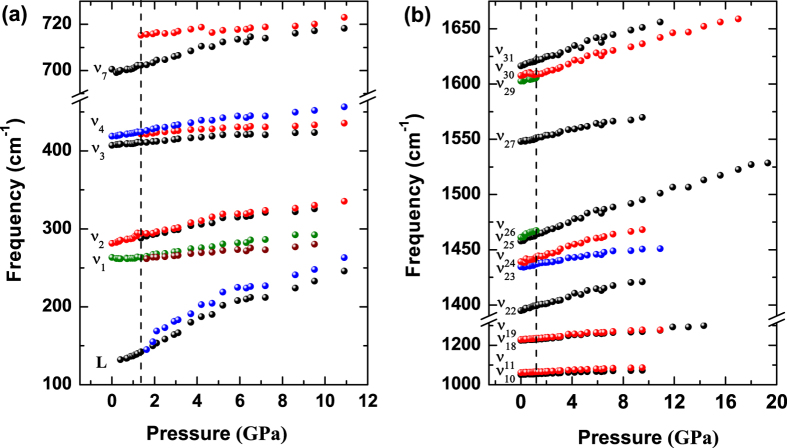
Pressure dependence of the vibrational frequencies for triphenylene at room temperature. (**a**) the changes of low frequencies; (**b**) the changes of high frequencies. The vertical dotted lines located at near 1.4 GPa indicate possible phase boundaries.

Figure 2(a,b) present the pressure dependence of the obtained band frequencies of triphenylene deduced from the spectral deconvolution. Upon compression to 1.4 GPa, there are many changes with some modes splitting, appearance and disappearance. It may be associated with the changes of crystal structure or molecular configuration, which will be further confirmed by synchrotron XRD measurements. When the pressure is further increased, the mode *ν*_1_ centered on 263 cm^−1^ shows a slope change and starts to shift to higher frequency. This anomaly seems to be accompanied by a splitting into overlapping components, mainly through the increasing peak width. More interestingly, the lattice mode has a larger frequency shift than the intramolecular modes, which suggests that the lattice parameters will be compressed rapidly with increasing pressure where the van der Waals forces between molecules play a dominant role. The shoulder peak, corresponding to C-H out of plane bending, was observed to appear on the high-frequency side of *ν*_7_, also indicating the possible molecular configuration change above 1.4 GPa. Meanwhile, the modes of *ν*_10_, *ν*_11_, *ν*_18_ and *ν*_19_, associating with C-H in plane bending, show a smooth and stable blue-shift. Based on the change of vibrational modes, we can infer that the effects of pressure on the change of the arrangement of molecule is larger than the change of molecular shape. It is worth noting that the increasing rate of lattice modes are slowed down above 6.0 GPa, which may associate to structure change.

### Analysis of structural evolution with pressure

**Figure 3:**
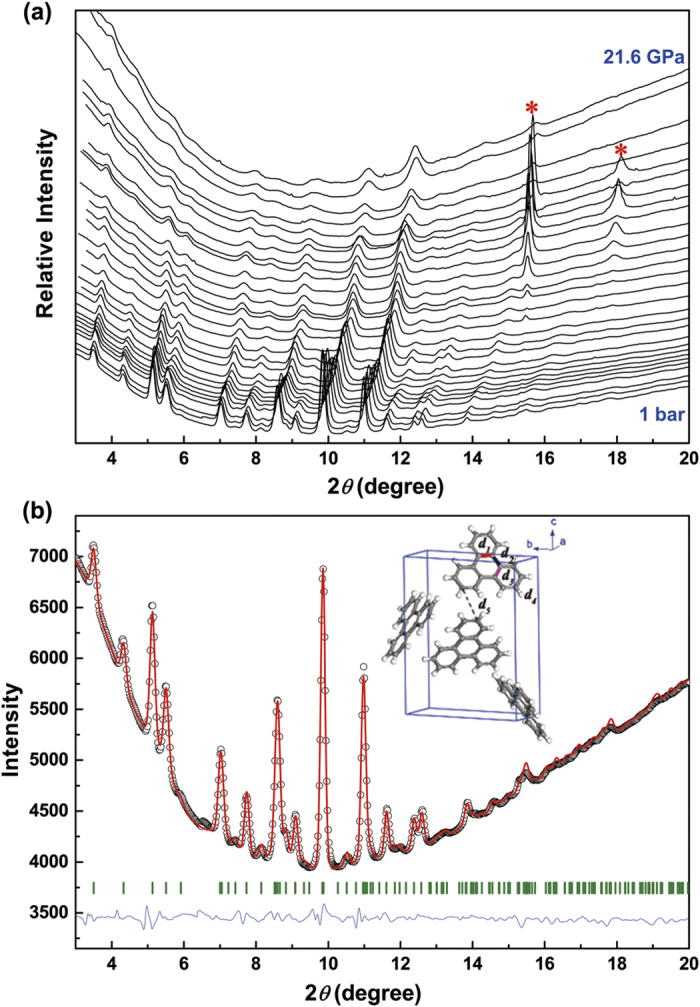
Structural properties of triphenylene under pressure. (**a**) Synchrotron x-ray diffraction patterns of triphenylene during the pressurization from ambient conditions to 21.6 GPa. The diffraction peaks of gold are marked with asterisks. (**b**) X-ray powder diffraction patterns of triphenylene at pressures of 0.2 GPa. The open circles represent the measured intensities and the red lines the results of profile refinements by the best Le Bail-fit with the space group of *P*2_1_2_1_2_1_. The positions of the Bragg reflections are marked by vertical lines and the difference profiles are shown at the bottoms (blue lines). The *R* values are *R*_*p*_ = 0.65%, *R*_*wp*_ = 0.48% for the fitting. The inset is the crystal and molecular structure of solid triphenylene.

**Figure 4:**
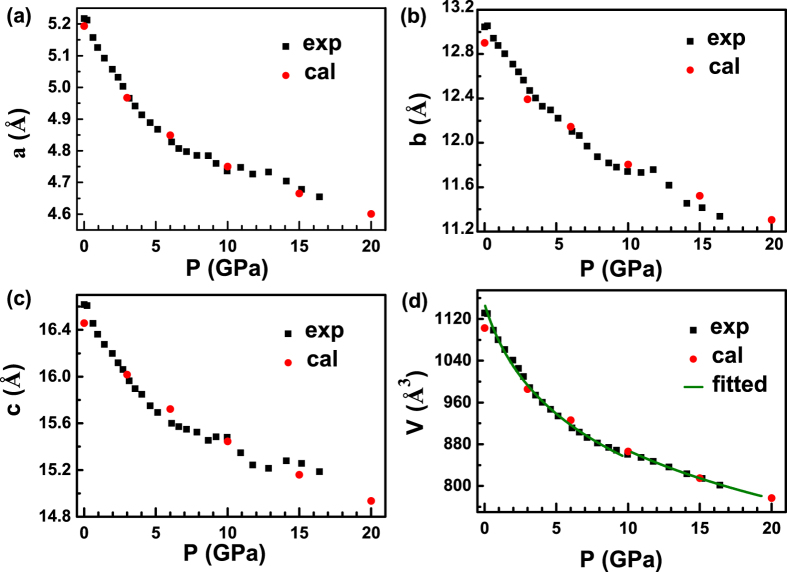
Lattice parameters *a* (**a**), *b* (**b**), *c* (**c**), and volume V (**d**) of triphenylene as a function of pressure. The black squares represent experimental data, and the red dots denote results from calculations. Solid line correspond to the result of a least-squares fit using a Murnaghan equation.

In order to explore the high-pressure structural properties of triphenylene, high-pressure XRD measurements were performed up to 21.6 GPa. Figure 3(a) shows some representative XRD patterns collected under various pressures at room temperature. It is clear that there are no dramatic changes in peak intensity, number, and sharpness of peaks, indicating no pressure-induced structural transitions. When looking at the evolution of the diffraction patterns as a function of applied pressure, the peak positions uniformly shift to higher angles (smaller *d*-spacing) as the crystal structure is compressed. From the results of XRD, we can confirm that the obvious changes at 1.4 GPa observed from Raman spectra is due to the change of molecular configuration. The results of measurement together with the structure refinement for 0.2 GPa, shown in Fig. 3(b), and the inset shows the crystal structure and the arrangement of molecular triphenylene. Here, the full refinements of diffraction patterns up to 16.4 GPa using the Le Bail method with GSAS software were carried out to obtain the lattice parameters at high pressures. The lattice parameters *a*, *b*, *c* and the volume V as a function of pressure at room temperature are plotted in Fig. 4. At pressure of 16.4 GPa the lattice constant *c* is reduced by 1.43 Å (−8.61%), while the lattice constants *a* and *b* have larger changes by 0.56 Å (−10.78%) and 1.71 Å (−13.09%), respectively. The slopes of three lattice parameters change with pressure above 10.0 GPa. The two lattice parameters *a* and *b* decrease rapidly, while the lattice parameter *c* becomes very flat with the increase of pressure, which results in a kink in the unit cell volume at approximately 10.0 GPa.

The pressure dependence of V is usually described by the three order Birch-Murnaghan equation of state (EOS)^22^, defined as


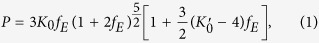


where 
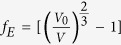
, *K*_0_ is the isothermal bulk modulus, 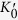 is the pressure derivative (=*dK*_0_/*dP*), and *V*_0_ is the volume of the unit cell at zero pressure. Fitting our data points, we obtained the values of *V*_0_ = 1144.0 ± 0.9 Å^3^, *K*_0_ = 12.73 ± 0.007 GPa and 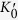 = 6.72 ± 0.003 for low-pressure region, and the fit to the data within the high pressure region results in values of *V*_0_ = 1134.0 ± 4.6 Å^3^, *K*_0_ = 17.83 ± 0.007 GPa and 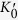 = 6.08 ± 0.007, where the value of ambient-pressure bulk modulus is slightly bigger than that of coronene^18^, indicating that the triphenylene is less compressible than coronene. The volume decreases by 29.14% in the pressure region studied. It has been reported^23^,^24^ that anthracene as one of the PAHs, a straight-chain aromatic hydrocarbon with three benzene rings, is decreased by 28% only from ambient pressure up to 10 GPa and decreases by 36.8% from ambient pressure to high pressure of 22.7 GPa in the unit cell volume, which suggests that triphenylene has relatively stronger var der Waals interaction or harder bonds than anthrancene. This difference is possibly due to the different arrangement of molecules, or the inherent properties of triangular molecular shape of triphenylene. For picene^21^, ~20% compression of the unit cell volume upon compression up to 8 GPa was observed, which is similar to the result of triphenylene.

In an effort to obtain more details of the crystal structure under pressure, we have performed the first-principles DFT calculations employing the VASP. We firstly optimized the geometrical structure of triphenylene and relaxed the inner atomic coordinates with the different functional forms. Comparing with experimental results, we found that the GGA leads to the enlargements of all of crystal parameters, meanwhile, the optimized crystal parameters based on LDA are correspondingly smaller than the experimental ones. Considering van der Waals interaction in version of vdWDF, the calculation results are in good agreement with the experimental values shown in Fig. 4, indicating the important role of van der Waals interaction in these systems. Previously, the pristine phenanthrene has been optimized with the different functional forms. It was found that the optimization results are consistent with the experimental results by considering vdWDF interaction^25^. This suggests that the application of such an approximation for the description of organic molecular crystals within vdWDF is valid.

**Figure 5:**
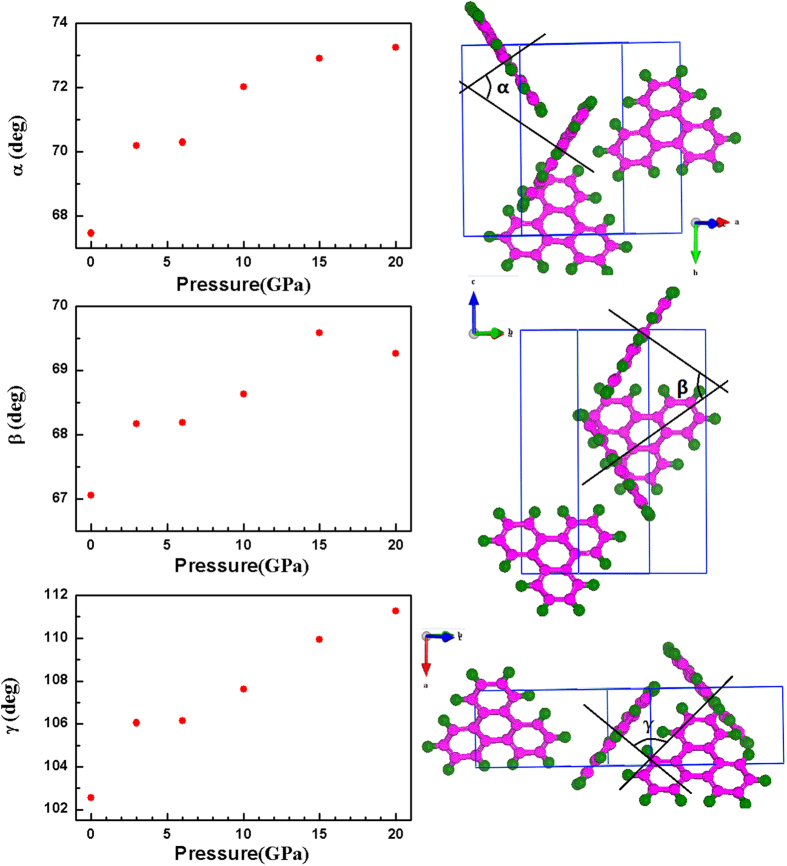
Left, the pressure dependence of the three angles. Right, the three angles *α*, *β*, and *γ* describe the orientation of the triphenylene molecules within the crystal structure.

The arrangement of the triphenylene molecules within the crystal structure is described by three angles *α*, *β*, and *γ*, which define the orientation of the molecular inside the unit cell. There are four molecules in one unit cell (see Fig. 3), one locats in the center, the other three molecules cross the three axis, respectively. The angle *α* defines between normal vectors (one vector perpendicular to molecular plane located in the center, the other vector perpendicular to molecular plane through *a* axis) of neighboring molecules. Similarly, the angle between normal vectors (one vector perpendicular to molecular plane located in the center, the other vector perpendicular to molecular plane through *b* axis) of neighboring molecules is named as *β*. The angle between normal vectors (one vector perpendicular to molecular plane located in the center, the other vector perpendicular to molecular plane through long molecular axis *c*) of neighboring molecules is denoted by *γ*. The pressure dependence of these angles and the definitions of these angle are given in Fig. 5. The three angles are determined from the calculation results, their values are *α* = 67.46°, *β* = 67.05°, and *γ* = 102.55° at ambient pressure. With increasing pressure, all three angles as a function of pressure have a kink at 6.0 GPa, coinciding with the change of lattice modes at about 6.0 GPa. Considering no pressure-induced structural transition, the anomaly of pressure-dependence of angles play main roles in the change of lattice modes at approximately 6.0 GPa. The biggest change of the angles is *γ*, which increases by 10°, however, the angles *α* and *β* show smaller dependences with pressure, with 4° and 3°, respectively. These angles increase with pressure, indicating that the molecular planes become more and more parallel relative to each other. It is can be interpreted that the van der Waals bonds play a more important role than interaction of intramolecular bonds in the low-pressure regions. Compared to the anthracene^23^,^24^, the arrangement of molecule of triphenylene is more dense in the unit cell, which corresponds to the small volume compressibility of solid triphenylene.

### Pressure-induced metallization

**Figure 6:**
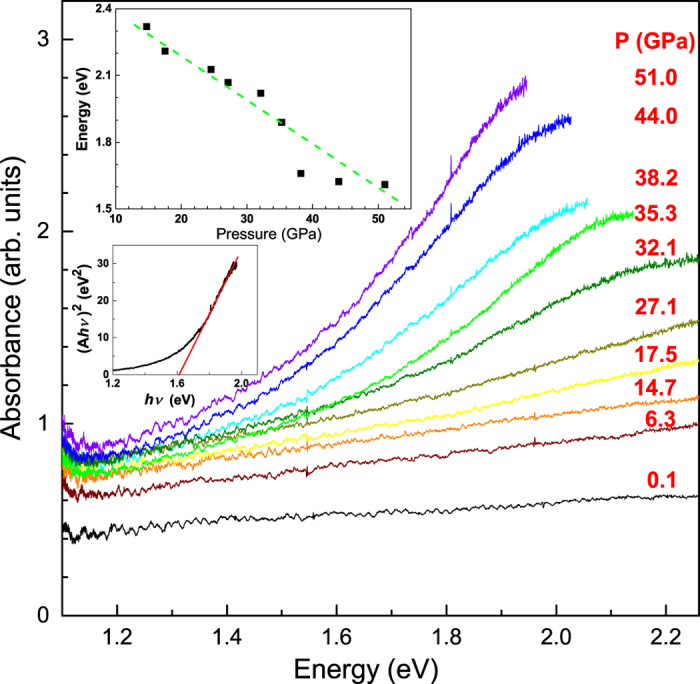
Optical absorption spectra of triphenylene in visible spectral range at different pressures and room temperature. The upper inset: the pressure dependence of the estimated band gap of triphenylene. The below inset shows the representative extrapolation for direct band gap at 51.0 GPa.

Optical measurements are of importance as a simple probe for exploring the novel phenomena related to the electronic states. The optical absorption spectra of triphenylene obtained at various pressures from ambient pressure to 51 GPa, are seen in Fig. 6. With increasing pressure, the absorption edge shifts to the lower-energy side, suggesting a decrease in the energy gap resulting from the changes of electronic structure. It is worth noting that above 17.5 GPa a strong band-gap feature appears in the spectra and shifts in red with pressure. At pressure of 44.0 GPa, the whole sample becomes dark in appearance. We estimated the band gap (*E*_*g*_) under the assumptions that the electronic transition is direct and the valance and conduction bands are parabolic. *E*_*g*_ is given by the intersection between the horizontal axis and a line obtained from the plot of (*Ahv*)^2^ versus *hv*. The results are shown in the inset of Fig. 6. *E*_*g*_ is observed at around 2.35 eV at 14.5 GPa. It shifts to lower energies with increasing pressure, and finally to approximately 1.6 eV at 51.0 GPa. The metallization of triphenylene is expected upon heavy compression.

The Raman scattering and synchrotron XRD measurements on triphenylene reveal the stable structural feature at high pressures. This is different to the case in coronene and phenanthrene in which structural transitions were observed^18^,^19^. Thus, tripheneylene is an appropriate material to examine the electric properties under pressure by performing DFT. The agreement between experiments and calculations for structural evolution indicates that we can continue to use the same structure and calculation method to explore the electronic properties at higher pressure, and the calculated results by considering vdDFT interaction are more reliable or credible.

Starting from ambient pressure, the simulation was done up to 250 GPa for solid triphenylene containing four formular units in a unit cell. At ambient pressure, the optimized lattice constants of *a* = 5.193 Å, *b* = 12.901 Å, and *c* = 12.901 Å are consistent with our experimental results and literature data^13^. With the increase of pressure, the lattice constants are reduced in three directions. Table 2 lists the variations of crystal lattice constants and several typical bond lengths under high pressure. We found that C-C, C-H bond lengths and intermolecular distance were all compressed as pressurizing. As a result, the large distortion and bending of triphenylene molecules were observed in the unit cell as pressure exceeds 180 GPa. Furthermore, the triphenylene molecules completely broke up above 200 GPa. However, the molecules were well preserved under the pressure of 180 GPa where no phase transition occurs.

**Figure 7:**
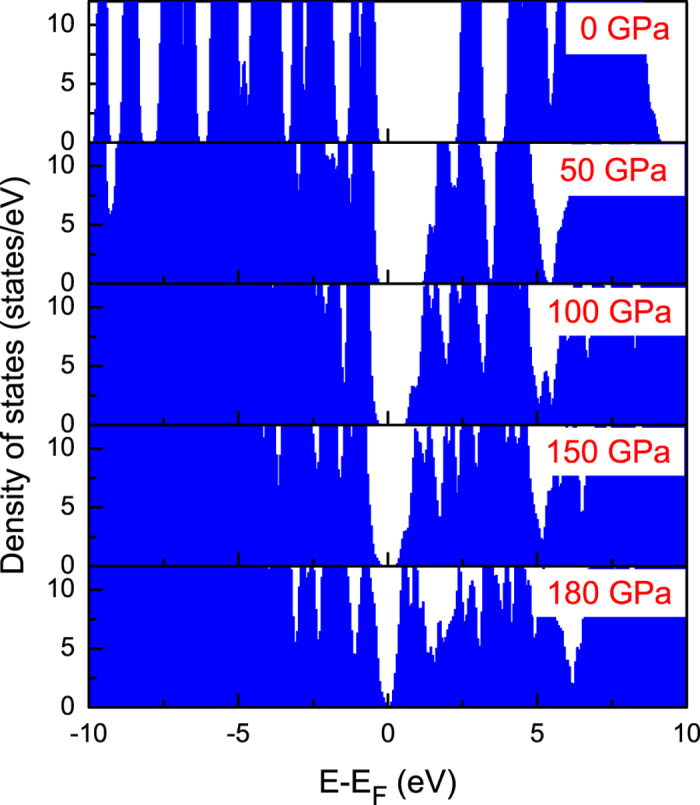
Calculated total density of states of triphenylene under pressure. E_*F*_ corresponds to Fermi level. The band gap obviously decreases with the increase of pressure.

The electronic structure calculations yield the evolution of the DOS with pressure (Fig. 7). At ambient pressure, the calculated band gap is about 2.5 eV. Solid triphenylene is a semiconductor. With the increase of pressure, the band gap gradually decreases. When the pressure reaches to 50.0 GPa, the *E*_*g*_ from calculated predication is 1.5 eV, which is excellent agreement with the experimental measurements. Analyzing the crystal structure and bond lengths shown in Table 2, we learn that the application of pressure results in the hybridization interactions strengthening among C-C atoms and molecule-molecule. With a further increase of pressure, the bands become broadened, making that the band gap closes at 180 GPa. We thus obtain the metallization in solid triphenylene at 180 GPa. The superconductivity of this system may be occurred at enough high pressures and low temperatures.

## Discussion

Both the spectroscopic and crystallographic results confirm the change of molecular configuration at 1.4 GPa. According to the analysis of x-ray patterns, there is no evidence of structural transition up to 21.6 GPa. We found that the structure of triphenylene is more stable than some other polycyclic aromatic hydrocarbons, such as phenanthrene and coronene, which have obvious structural phase transitions under pressure. Upon compression to 10.0 GPa, the lattice parameters have abrupt changes, resulting in the different bulk moduli between the low-pressure and high-pressure regions. The rapid volume reduction between 0 and 10 GPa is attributable to the squeezing out of the van der Waals space between triphenylene units. Previous studies^19^ revealed that most polycyclic aromatic hydrocarbons have transformed into amorphous clusters at high pressures, association with the generation of D band and G band, which can be observed from Raman spectra. However, the Raman modes of triphenylene show a smooth and uniform blue-shift without abrupt changes. Combining with the XRD results, one can infer that the solid triphenylene maintains stable and intact at high pressures.

The high-pressure stability of triphenylene is similar to the organic compounds isoviolanthrone^26^. Isoviolanthrone shows a continuous drop in resistance with the application of pressure and then changes from insulator to semiconductor^27^. Meanwhile, the application of pressure has driven pentacene from insulator at ambient pressure to metal at high pressure. In this aspect, the optical absorption spectra measurements exhibit that the band gap is decreased with the increase of pressure, and reaches to 1.6 eV at 51.0 GPa. Meanwhile, the DFT calculation predicts that the bands were found to broaden with the increase of pressure, and the band gap is decreased to 1.5 eV at 50.0 GPa, and finally it exhibits a metallic character with the closed band gap at 180 GPa. Upon compression, benzene displays a possible intermediate metallic phase at 190 GPa, and the band gap opens up again above 210 GPa^12^. Both the triphenylene and benzene belong to PAHs. These compounds have similar structure and electronic behavior at high pressures. Our results indicate that triphenylene remains substantially a molecular solid up to 180 GPa, and may keep unsaturated molecule. The achievement of metallization in triphenylene may be due to the more stable structure than other polycyclic aromatic hydrocarbons under pressure.

## Conclusion

We have reported the optical and structural properties of triphenylene at high pressures by combining Raman scattering, XRD, and visible transmission spectroscopy measurements. Although the substantial changes in the molecular configuration is observed at 1.4 GPa from Raman spectra, the x-ray patterns indicate no pressure-induced structural transitions. DFT calculations reproduce an excellent agreement with experimental data. The three orientation angles show different changes with pressure due to the different compression ratios along three axes. The neighboring molecules are found to become more and more parallel relative to each other. The optical absorption spectra indicate that the band gap is gradually decreased with increasing pressure. Pressure-induced increase of the intermolecular overlap results in a vanishing of the band gap at 180 GPa, yielding a pressure-induced metallization. Our findings represent a significant step toward the understanding of the high pressure behavior of polycyclic aromatic hydrocarbons. Measurements of transport properties are on the way to examine pressure-induced metallization in this studied hydrocarbon.

## Methods

Triphenylene (99% purity) was purchased from TCI Co. and chosen for experiments without further purification. A diamond anvil cell was used with anvils in 300 *μ*m culet for Raman spectroscopy and optical absorption spectroscopy experiments at room temperature. A stainless steel gasket with a drilled hole of ~100 *μ*m in diameter was used as the sample chamber. The pressure was determined from the shift of the luminescence spectrum of a ruby chip enclosed in the sample^28^. Renishaw Invia Raman system with a spectrometer (with 2400 lines/mm grating) equipped with a di-monochromator and a charge coupled device detector was adopted for the measurements, achieving a spectral resolution of below 1 cm^−1^. The Raman spectra were measured in backscattering geometry with visible laser excitation (532 nm). In order to avoid damaging the sample, we used 5% laser power to collect data. UV-VIS fiber light source L7893 series was adopted in our absorption spectroscopy measurement, the white-light source covering a wide spectra range was achieved by the combination of a deuterium lamp and a tungsten lamp, the spectrum could cover the energy range from 1.0 eV to 3.0 eV.

Synchrotron XRD was carried out via the angle dispersive experiments at the 15U1 beamline at the Shanghai Synchrotron Radiation Facility (SSRF). Silicone oil was used as pressure transmitting medium to maintain quasi-hydrostatic pressure environment in the studied pressure range. Both a chip ruby and gold were loaded to monitor the pressures. The measurements were performed using a wavelength of 0.62647 Å and the sample-to-detector distance and the image plane orientation angle were calibrated with standard CeO_2_ powder diffraction. The two-dimensional diffraction images were converted to 2*θ* versus intensity data plots using the FIT2D software.

The DFT calculations employed are based on the plane wave/pseudo potential approach using the Vienna *ab* initio simulation package (VASP)^29^ employing the projector augmented wave (PAW) and Perdew- Burke-Ernzerhof (PBE) exchange. A conjugate-gradient algorithm was used to relax the ions into their instantaneous ground state and the wave function has been expanded in a plane-wave basis up to cutoff of 600 eV. The Monkhorst-Pack k-point grids were generated according to the specified k-point separation 0.03 Å^−1^. Relaxation of the electronic degrees of freedom was stopped when the total energy between two steps by less than 10^−1^. The local density approximation (LDA), the generalized gradient approximation (GGA) of PBE version and the van der Waals density functional (vdW-DF2)^30^ were adopted to describe the electronic exchange-correlation interactions to the optimization of crystal structures. The self-consistent properties such as charge density and DOS were calculated by employing the highly precise full-potential linearized augmented plane wave (FLAPW) method.

## Additional Information

**How to cite this article**: Zhao, X.-M. *et al.* Combined experimental and computational study of high-pressure behavior of triphenylene. *Sci. Rep.* **6**, 25600; doi: 10.1038/srep25600 (2016).

## Figures and Tables

**Table 1:** The Raman modes, proposed assignments (from ref. 13), and observed wave number of triphenylene.

Raman modes	Assignment	Obs. (cm^−1^)	Raman modes	Assignment	Obs. (cm^−1^)
L_1_	lattice mode	132	*ν*_16_	C-H in plane Bending	1174
*ν*_1_	C-C-C out-of-plane Bending	263	*ν*_17_	C-H in plane Bending	1181
*ν*_2_	C-C-C out-of-plane Bending	282	*ν*_18_	C-H in plane Bending	1225
*ν*_3_	C-C-C out-of-plane Bending	407	*ν*_19_	C-H in plane Bending	1229
*ν*_4_	C-C-C out-of-plane Bending	419	*ν*_20_	C-H in plane Bending	1247
*ν*_5_	C-C-C out-of-plane Bending	543	*ν*_21_	Aromatic C-C stretching	1299
*ν*_6_	C-H out-of-plane Bending	619	*ν*_22_	Aromatic C-C stretching	1395
*ν*_7_	C-H out-of-plane Bending	701	*ν*_23_	Aromatic C-C stretching	1434
*ν*_8_	C-H out-of-plane Bending	771	*ν*_24_	Aromatic C-C stretching	1439
*ν*_9_	C-H out-of-plane Bending	776	*ν*_25_	Aromatic C-C stretching	1458
*ν*_10_	C-H in plane Bending	1052	*ν*_26_	Aromatic C-C stretching	1461
*ν*_11_	C-H in plane Bending	1062	*ν*_27_	Aromatic C-C stretching	1547
*ν*_12_	C-H in plane Bending	1085	*ν*_28_	Aromatic C-C stretching	1579
*ν*_13_	C-H in plane Bending	1159	*ν*_29_	Aromatic C-C stretching	1602
*ν*_14_	C-H in plane Bending	1163	*ν*_30_	Aromatic C-C stretching	1608
*ν*_15_	C-H in plane Bending	1171	*ν*_31_	Aromatic C-C stretching	1616

**Table 2:** Optimized crystal lattice constants under several pressures.

Pressure	*a* (Å)	*b* (Å)	*c* (Å)	*d*_1_(Å)	*d*_2_(Å)	*d*_3_(Å)	*d*_4_(Å)	*d*_5_(Å)
0 GPa	5.193	12.901	16.458	1.428	1.469	1.427	1.082	3.774
50 GPa	4.370	10.532	14.081	1.379	1.405	1.370	1.056	2.788
100 GPa	4.170	9.966	13.180	1.347	1.368	1.338	1.043	2.534
150 GPa	4.060	9.644	12.498	1.325	1.339	1.318	1.030	2.328
180 GPa	3.956	9.561	12.069	1.312	1.309	1.316	1.024	2.325

As shown in Fig. 3 *d*_1_, *d*_2_, and *d*_3_ are defined as three kinds of C-C bond lengths, *d*_4_ is the C-H bond length, while the *d*_5_ marks the intermolecular distance.
